# Preoperative serum cystatin-C as a potential biomarker for prognosis of renal cell carcinoma

**DOI:** 10.1371/journal.pone.0178823

**Published:** 2017-06-06

**Authors:** Shengjie Guo, Yunfei Xue, Qiuming He, Xiaobo He, Kunbin Guo, Pei Dong, Kai Yao, Guangwei Yang, Dong Chen, Zaishang Li, Xiangdong Li, Zike Qin, Zhuowei Liu, Wenjie Cheng, Chao Guo, Meng Zhang, Hui Han, Fangjian Zhou

**Affiliations:** 1 Department of Urology, Sun Yat-Sen University Cancer Center, State Key Laboratory of Oncology in South China, Collaborative Innovation Center for Cancer Medicine, Guangzhou, China; 2 Medicine school of Sun Yat-Sen University, Guangzhou, China; 3 Department of Medical Oncology, the Fifth Affiliated Hospital of Sun Yat-Sen University, Zhuhai, China; 4 Institute of Clinical Pharmacology, Guangzhou University of Chinese Medicine, Guangzhou, China; 5 Department of Urology, the Fifth Affiliated Hospital of Sun Yat-Sen University, Zhuhai, China; 6 The Jiangcheng Branch of Yangjiang People’s Hospital, Yangjiang, Guangdong, China; Icahn School of Medicine at Mount Sinai, UNITED STATES

## Abstract

**Purpose:**

The prognostic value of serum cystatin-C (Cys-C) in renal cell carcinoma (RCC) remains unknown. The purpose of this study is to explore the prognostic value of Cys-C for RCC patients.

**Patients and methods:**

The levels of preoperative Cys-C, creatinine (CRE) and estimated glomerular filtration rate (e-GFR) were retrospectively collected in 325 RCC patients undergoing surgery. The cutoff values of Cys-C, CRE and e-GFR were determined by the standardized Cutoff Finder algorithm. The receiver operating characteristic (ROC) curve and pairwise comparison were performed to compare the three variables. Univariate and multivariate Cox regression analyses were performed to investigate the prognostic value of serum Cys-C in RCC.

**Results:**

Based on the analysis of Cutoff Finder algorithm, ROC curve and pairwise comparison, the preoperative Cys-C was superior to CRE and e-GFR as a predictive factor in RCC. Multivariate Cox regression analyses showed that high preoperative Cys-C (>1.09 mg/L) was significantly associated with shorter overall survival (OS) in all RCC patients (hazard ratio [HR], 1.59; P = 0.012), patients at pT1-2 (P<0.001), pN0 (P<0.001) and pM0 stages (P<0.001). Moreover, Multivariate Cox regression analyses also showed that in the 306 patients without metastasis, high preoperative Cys-C was also associated with shorter disease-free survival (DFS) (HR, 3.50; P = 0.013).

**Conclusions:**

An elevated preoperative Cys-C level was demonstrated to be related with worse survival in patients with RCC. Measuring preoperative serum Cys-C might be a simple way for finding poor prognostic patients and patients with elevated preoperative Cys-C level should be more closely followed up.

## Introduction

Renal cell carcinoma (RCC) is one of the most common malignant urogenital tumors [1, 2]. In recent years, RCC can be detected more frequently at early stage because of increased use of imaging techniques including ultrasound and computed tomography (CT) [3, 4]. Despite more RCC has been diagnosed at early stage, the disease death rate is still rising. Approximately 20~30% of patients with localized disease after radical or partial nephrectomy will later develop metastatic disease [5] due to lack of curative therapies for metastatic RCC. Majority of patients at advantageous stage will die from cancer. There is an urgent need for prognostic factor to predict high risk of recurrent or metastatic patients who will be closely followed up. Although TNM [6] and Fuhrman’s nuclear grading systems [7] are most useful prognostic factors, they are still not perfect [8]. Other well-known prognostic factors include lymphocytic infiltration, necrosis and histological subtype [8]. Due to the insufficiency of these prognostic factors, new factors including clinical and laboratory indicators have started to be considered.

Cystatin-C (Cys-C) is a cysteine protease inhibitor produced by nearly all nucleated cells and excreted into the bloodstream [9]. Cys-C has multiple biological functions including controlling extracellular proteolysis via inhibition of cysteine peptidases, modulating immune system and exerting antibacterial and antiviral activities [9, 10]. Cys-C is freely filtered by the glomerulus, and reabsorbed and metabolized by the proximal tubules. Therefore, it is considered an accurate endogenous marker of GFR in various types of kidney diseases [11, 12]. As a secreted cysteine protease inhibitor, Cys-C may inhibit cathepsins (B, D, H, L and S) and other human lysosomal cysteine proteases [13]. By inactivating cathepsin protease activity, Cys-C is served as an inhibitor of cancer cell invasion and metastasis [14, 15]. Abnormal serum levels of Cys-C or cathepsin B/cystatin C complex have been suggested as diagnostic and prognostic indicators for cancers of skin, breast, colon and lung [16].

However, little is known about the expression of Cys-C in RCC. The purpose of this research was to analyze the prognostic value of serum Cys-C in patients with surgical RCC.

## Patients and methods

### Patients

398 patients who were diagnosed with RCC and treated with resection of primary tumor at Sun Yat-sen University Cancer Center (SYSUCC) between September 2009 and January 2013 were retrospectively enrolled in the study. To ensure that data were collected objectively and accurately, patient inclusion criteria were Age≥18 years, and no previous or coexisting tumor. The following exclusion criteria were used: patients with a history of anticancer therapy, or other concurrent concomitant diseases (including diabetes, uncontrolled hypertension, inflammation, and infection), or insufficient biochemical test results, or survival data. Among them, 325 patients were enrolled in this study. The study was approved by the Institutional Review Board of SYSUCC and performed in accordance with the ethical standards of the World Medical Association Declaration of Helsinki. All included patients provided written informed consent and their information were recorded and registered in our cancer registry system. The authenticity of this article has been validated by uploading the key raw data onto the Research Data Deposit public platform (www.researchdata.org.cn), with the approval RDD number as RDDA2017000174.

### Patients follow-up

Follow-up evaluations were carried out included physical, laboratory examination and radiological examinations referring to the National Comprehensive Cancer Network (NCCN) clinical practice guidelines. In addition, all patients were also followed up via telephone interviews. The last follow-up was completed in November 1, 2015, and after that, the whole data was analyzed. The primary endpoint was overall survival (OS), which was defined as the interval between surgery and last follow-up or death from all causes. The secondary endpoint was disease free survival (DFS), which was calculated as the interval between surgery and last follow-up or recurrence.

### Statistical analysis

Continuous variables and categorical variables were presented as median (range), and number (percentage), respectively. Percentage differences between groups were compared with χ2 test or Fisher's exact test. Continuous data were compared using Mann-Whitney test. Relationship between variables was determined using two-sided Spearman’s correlation coefficient. Estimated glomerular filtration rate (e-GFR) was calculated by the Cockcroft-Gault [17] adjusted for body surface area by normalizing the output per 1.73 m^2^ of body surface area. The optimal cutoff values of preoperative serum Cys-C, creatinine (CRE) and e-GFR were determined using a web-based R software engineered and designed by Budczies et al [18] (http://molpath.charite.de/cutoff/). The predictive value of the established model was assessed using the area under the receiver operating characteristic (ROC) curve (AUC), and the pairwise comparison of AUC values of significant biomarkers was carried out using z statistical method. The associations of preoperative serum Cys-C with pT-stage, pN-stage and pM-stage were assessed using non-parametric analysis of variance (ANOVA). OS and DFS were measured using Kaplan-Meier curves and log-rank test. Variables with prognostic significance for survival were identified using univariate Cox regression analyses and further analyzed using multivariate Cox regression analysis to test their independence. Hazard ratios (HRs) estimated from the Cox analysis were reported as relative risks with corresponding 95% confidence intervals (CIs). All statistical analyses were performed using SPSS21.0 software (IBM, Armonk, NY) and MedCalc (MedCalc Software, Ostend, Belgium). All tests were two-sided and a *P* value <0.05 was considered statistically significant.

## Results

### Clinicopathologic characteristics

The 325 enrolled patients had mean age of 51 years old. Among them, 222(68.30%) were males. In addition, 260(80.00%), 20(6.20%) and 45(13.80%) were pathologically diagnosed as clear cell carcinoma, papillary type and other types, respectively; 227 (69.80%), 46 (14.2%), 26 (8.00%) and 26 (8.00%) were staged in I, II, III and IV, respectively. **Table 1** shows their baseline characteristics.

**Table 1:** Clinicopathological characteristics of patients.

Characteristics	Value
Age, (years) (median)	51
Range	18–84
Gender	
Male	222 (68.30%)
Female	103 (31.70%)
BMI, (kg/m^2^) (median)	23.44
Range	15.41–38.76
Fuhrman grade	
Ⅰ	47 (14.50%)
Ⅱ	156 (48.00%)
Ⅲ	37 (11.40%)
Ⅳ	7 (2.20%)
unknown	78 (24.00%)
Pathological types	
Clear cell carcinoma	260 (80.00%)
Papillary carcinoma	20 (6.20%)
Others	45 (13.80%)
pTNM stage	
Ⅰ	227 (69.80%)
Ⅱ	46 (14.20%)
Ⅲ	26 (8.00%)
Ⅳ	26(8.00%)
pT-stage	
pT1	235 (72.30%)
pT2	53 (16.30%)
pT3	26 (8.00%)
pT4	11 (3.40%)
pN-stage	
pN0	305 (93.80%)
pN1	20 (6.20%)
pM-stage	
pM0	306 (94.20%)
pM1	19 (5.80%)
Preoperative ALP, (U/L) (median)	71.90
Range	16.00–419.00
Preoperative TP, (g/L) (median)	72.67
Range	29.65–93.46
Preoperative UA, (μmol/L) (median)	358.30
Range	112.60–616.30
Preoperative CRE, (μmol/L) (median)	75.40
Range	38.10–221.90
Preoperative Cystatin-C, (mg/L) (median)	0.98
Range	0.50–2.96
Preoperative e-GFR, (mL/min/1.73 m^2^) (median)	78.69
Range	16.31–144.31

Abbreviation: BMI: body mass index; ALP: alkaline phosphatase; TP: total protein; UA: uric acid; CRE: creatinine; e-GFR: estimated glomerular filtration rate.

The mean follow-up time was 48.74 months. Mean DFS and OS were 67.25 and 66.66 months, respectively.

### The relationship of serum Cys-C, CRE or e-GFR with predictor of prognosis of RCC patients

**Fig 1:**
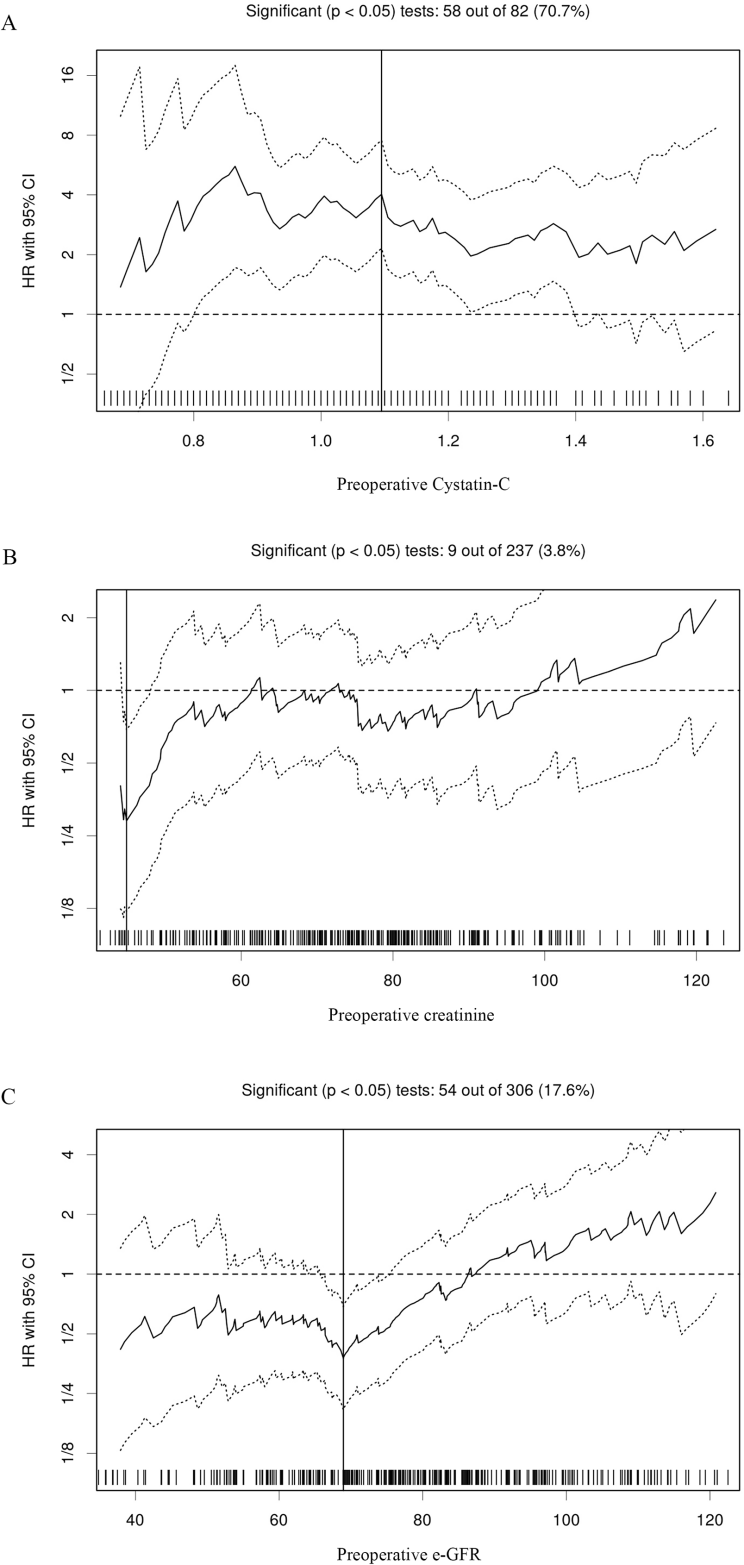
Hazard ratios and cutoff values of independent factors for overall survival of patients with renal cell carcinoma. (A) Preoperative serum cystatin C; (B) Creatinine (CRE); and (C) estimated glomerular filtration rate (e-GFR) (C). The vertical line designates the optimal cutoff values with the most significant (log-rank test) split.

Serum Cys-C is considered a potential marker of renal function, thus, its relationship with serum CRE and e-GFR were analyzed for predicting the prognosis of RCC patients. The results showed that preoperative serum CRE and e-GFR levels were positively and negatively correlated to Cys-C as determined by Spearman’s correlation coefficient test (r = 0.365, P<0.001; and r = -0.416, P<0.001, respectively). In addition, preoperative CRE and e-GFR levels were negatively correlated (r = -0.718, P<0.001). Analysis using the Cutoff Finder showed the recommended cutoff values of preoperative serum Cys-C, CRE and e-GFR for evaluating OS were 1.09, 44.9 and 77.16, respectively **(Fig 1)**. In addition, ROC curve analysis of the three indicators showed that preoperative serum Cys-C was significant (AUC = 0.69, P<0.001), but not CRE (AUC = 0.52, P = 0.594) and e-GFR (AUC = 0.55, P = 0.325) **(Table 2)**. Furthermore, pairwise comparisons of the three biomarkers showed that preoperative serum Cys-C is a better index than CRE and e-GFR for assessing the prognosis of RCC patients **(Table 3)**.

**Table 2:** The analysis of three variables according to the receiver operating characteristic curve.

Variable	specificity	sensitivity	Optimal cutoff value	AUC	The low 95% CI	The high 95% CI	P value
Preoperative Cystain-C	0.71	0.65	1.09	0.69	0.64	0.74	<0.001
Preoperative CRE	0.96	0.13	44.70	0.52	0.47	0.58	0.594
Preoperative e-GFR	0.71	0.53	68.94	0.55	0.49	0.60	0.325

**Table 3:** The pairwise comparison for the three variables.

Preoperative Cystain-C **vs.** Preoperative CRE	
Difference between areas	0.170
Standard Error	0.078
95% CIs	0.016–0.323
Z statistic	2.167
Significance level	P = 0.030
Preoperative Cystain-C **vs.** Preoperative e-GFR	
Difference between areas	0.143
Standard Error	0.047
95% CIs	0.048–0.236
Z statistic	2.979
Significance level	P = 0.002
Preoperative CRE **vs.** Preoperative e-GFR	
Difference between areas	0.027
Standard Error	0.096
95% CIs	-0.162–0.217
Z statistic	0.281
Significance level	P = 0.778

Overall, based on the cutoff value of serum Cys-C, these patients were divided into low serum Cys-C (≤1.09 mg/L) and high serum Cys-C (>1.09 mg/L) groups, respectively.

### The relationship between clinicopathological characteristics and preoperative serum Cys-C

**Fig 2:**
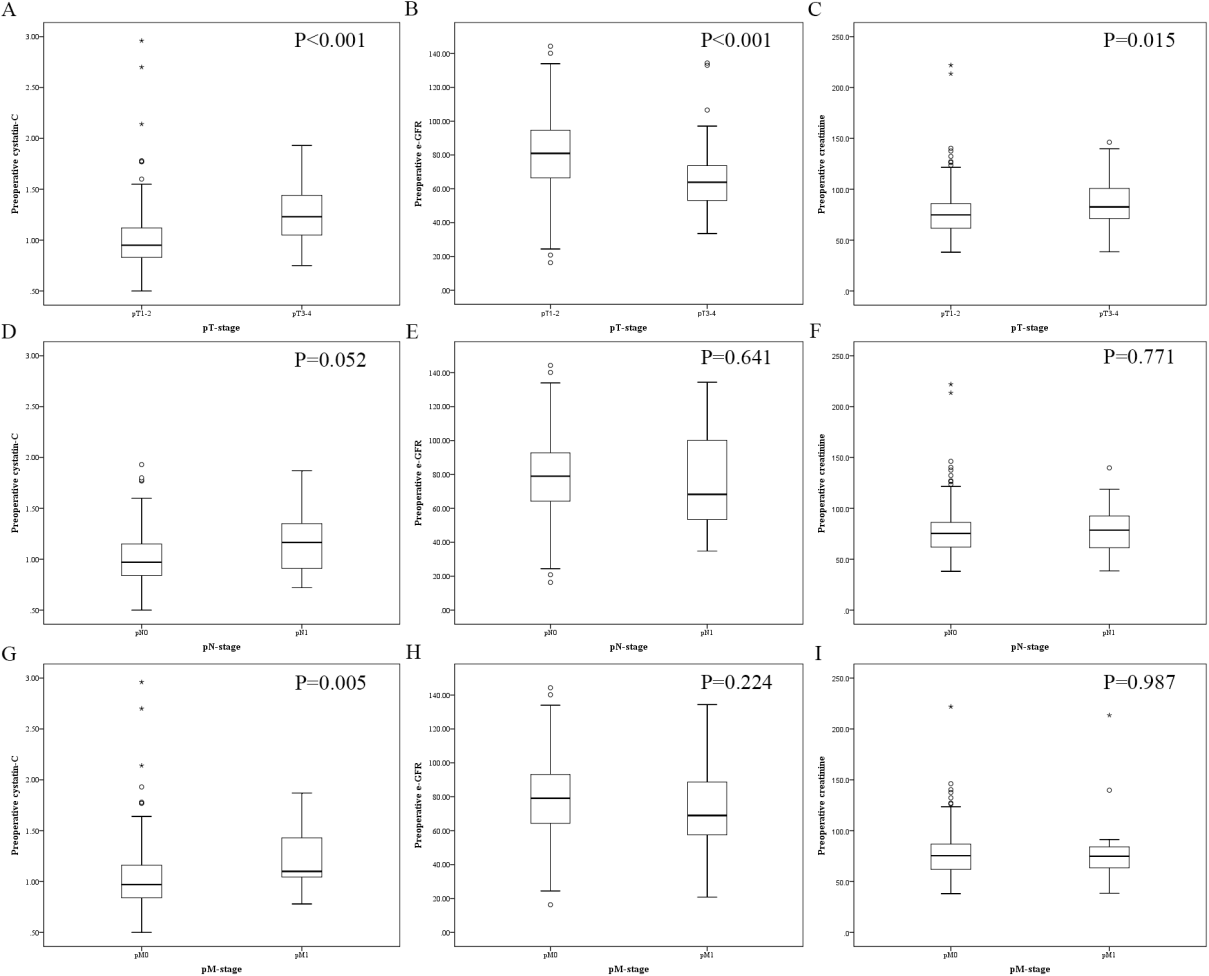
Box plot diagrams showing the level of preoperative cystatin C, estimated glomerular filtration rate (e-GFR) and creatinine (CRE) in different pT-status, pN-status, and pM-status. (A) Cystatin C in pT1-2 and pT3-4 RCC patients, (B) e-GFR in pT1-2 and pT3-4 RCC patients, (C) CRE in pT1-2 and pT3-4 RCC patients, (D) Cystatin C in pN0 and pN1 RCC patients, (E) e-GFR in in pN0 and pN1 RCC patients, (F) CRE in pN0 and pN1 RCC patients, (G) Cystatin C in pM0 and pM1 RCC patients, (H) e-GFR in pM0 and pM1 RCC patients, (I) CRE in pM0 and pM1 RCC patients.

The clinicopathological characteristics in each subgroup are described in **Table 4**. Patients with low preoperative serum Cys-C were significantly younger (P<0.001) and had lower preoperative ALP, CRE and UA levels but higher preoperative e-GFR. In addition, preoperative serum Cys-C level was associated with gender (P = 0.008), pT-stage (P<0.001), pN-stage (P = 0.036), pM-stage (P = 0.020), pTNM-stage (P<0.001), Fuhrman grade (P = 0.005) and pathological types (P = 0.016), but not BMI (P = 0.592) and preoperative TP (P = 0.481). Moreover, as a continuous variable, preoperative serum Cys-C level was significantly higher in RCC patients at pT3-4 and pM1 than RCC patients at pT1-2 (P<0.001) and pM0 (P = 0.005), respectively, but similar among RCC patients at different pN-stage (P = 0.052). In addition, the preoperative e-GFR level was lower in patients with pT3-4 stage than pT1-2 stage (P<0.001) and the preoperative CRE level was higher in patients with pT3-4 stage (P = 0.015). The preoperative CRE and e-GFR levels were not statistically significant for the subgroup of the pN and pM stage. The details were depicted in **Fig 2**.

**Table 4:** Clinicopathological variables of patients stratified by preoperative serum cystatin-C.

Characteristics	Cystatin-C≤1.09 mg/L (n = 216)	Cystatin-C>1.09 mg/L (n = 109)	P-value
Age, (years)	49.00 (18.00–78.00)	56.00 (25.00–84.00)	<0.001 ^a^
BMI, (kg/m^2^)	23.44 (16.02–35.67)	23.62 (15.41–38.76)	0.592 ^a^
Gender			0.008 ^b^
Male	137 (63.40%)	85 (78.00%)	
Female	79 (36.60%)	24 (22.00%)	
pT-stage			<0.001 ^b^
pT1	175 (81.00%)	60 (55.00%)	
pT2	30 (13.90%)	23 (21.10%)	
pT3	8 (3.70%)	18 (16.50%)	
pT4	3 (1.40%)	8 (7.30%)	
pN-stage			0.036 ^b^
pN0	207 (95.80%)	98 (89.90%)	
pN1	9 (4.20%)	11 (10.10%)	
pM-stage			0.020 ^b^
pM0	208 (96.30%)	98 (89.90%)	
pM1	8 (3.70%)	11 (10.10%)	
pTNM-stage			<0.001 ^b^
Ⅰ	171 (79.20%)	56 (51.40%)	
Ⅱ	27 (12.50%)	19 (17.40%)	
Ⅲ	8 (3.70%)	18 (16.50%)	
Ⅳ	10 (4.60%)	16 (14.70%)	
Fuhrman grade			0.005 ^b^
Ⅰ	36 (16.70%)	11 (10.10%)	
Ⅱ	103 (47.70%)	53 (48.60%)	
Ⅲ	16 (7.40%)	21 (19.30%)	
Ⅳ	3 (1.40%)	4 (3.70%)	
Unknown	58 (26.90%)	20 (18.30%)	
Pathological types			0.016 ^b^
Clear cell carcinoma	163 (75.50%)	97 (89.00%)	
Papillary carcinoma	16 (7.40%)	4 (3.70%)	
Others	37 (17.10%)	8 (7.30%)	
Preoperative ALP (U/L)	70.90 (16.00–237.30)	77.90 (27.50–419.00)	0.002 ^a^
Preoperative TP (g/L)	72.67 (29.65–93.46)	72.75 (56.82–85.09)	0.481 ^a^
Preoperative CRE (μmol/L)	70.85 (38.10–123.60)	84.60 (40.80–221.90)	<0.001 ^a^
Preoperative UA (μmol/L)	347.95 (120.70–589.60)	378.70 (112.60–616.30)	0.008 ^a^
Preoperative e-GFR (mL/min/1.73 m^2^)	83.33 (35.82–144.31)	65.66 (16.31–131.90)	<0.001 ^a^

Abbreviation: BMI: body mass index; ALP: alkaline phosphatase; TP: total protein; UA: uric acid; CRE: creatinine; e-GFR: estimated glomerular filtration rate.

^a^ Kraskal-Wallis test

^b^ Chi-square test

### The relationship of preoperative serum Cys-C with OS of RCC patients

Univariate Cox proportional hazards regression model analysis based on the cut-off levels of serum Cys-C showed that low preoperative serum Cys-C was associated with better OS (P<0.001). In addition, BMI, Fuhrman-grade, pTNM stage, pT-status, pN-status, pM-status and ALP were also remained clinically and statistically significant predictors of prognosis (**Table 5**). Multivariate Cox regression analysis showed that low serum Cys-C was a significant independent predictor favorable for OS (HR, 1.59; P = 0.012). In addition, pN-status and pM-status also remained clinically and statistically significant predictors of prognosis (**Table 5**).

**Table 5:** Univariate and multivariate analyses in 325 patients with RCC for overall survival (OS).

	Univariate Analysis			Multivariate Analysis		
Variables	HR	95%CI	P value	HR	95%CI	P value
Age, (years) continuous	1.01	0.99 to 1.04	0.338 ^a^			
Gender						
Male	1.00 (ref.)					
Female	1.15	0.62 to 2.16	0.656 ^a^			
BMI	0.86	0.78 to 0.94	0.001 ^a^	0.92	0.83 to 1.02	0.140 ^b^
Fuhrman grade						
Ⅰ	1.00 (ref.)			1.00 (ref.)		
Ⅱ	2.33	0.53 to 10.19	0.261 ^a^	1.73	0.38 to 7.80	0.473 ^b^
Ⅲ	8.41	1.86 to 38.02	0.005 ^a^	2.97	0.59 to 14.92	0.186 ^b^
Ⅳ	16.43	3.01 to 89.79	0.001 ^a^	3.22	0.41 to 24.77	0.260 ^b^
unknown	3.59	0.80 to 16.21	0.096 ^a^	1.70	0.35 to 6.16	0.501 ^b^
Pathological types						
Clear cell carcinoma	1.00 (ref.)					
Papillary carcinoma	2.23	0.87 to 5.72	0.095 ^a^			
Others	0.92	0.36 to 2.36	0.870 ^a^			
pTNM-stage						
Ⅰ	1.00 (ref.)					
Ⅱ	1.63	0.53 to 5.05	0.398 ^a^			
Ⅲ	7.72	3.25 to 18.32	<0.001 ^a^			
Ⅳ	22.61	10.80 to 47.37	<0.001 ^a^			
pT-stage						
pT1	1.00 (ref.)			1.00 (ref.)		
pT2	2.46	1.09 to 5.51	0.029 ^a^	1.28	0.53 to 3.08	0.573 ^b^
pT3	5.85	2.61 to 13.14	<0.001 ^a^	1.90	0.77 to 4.67	0.160 ^b^
pT4	14.18	6.08 to 33.09	<0.001 ^a^	0.85	0.23 to 3.10	0.813 ^b^
pN-stage						
pN0	1.00 (ref.)			1.00 (ref.)		
pN1	13.48	7.09 to 25.64	<0.001 ^a^	5.81	2.25 to 14.99	<0.001 ^b^
pM-stage						
pM0	1.00 (ref.)			1.00 (ref.)		
pM1	14.89	7.76 to 28.6	<0.001 ^a^	4.64	1.84 to 11.69	0.001 ^b^
Preoperative ALP, (U/L), continuous	1.01	1.00 to 1.01	<0.001 ^a^	1.00	0.99 to 1.01	0.696 ^b^
Preoperative TP, (g/L), continuous	1.02	0.97 to 1.07	0.386 ^a^			
Preoperative CRE, (μmol/L), continuous	1.00	0.99 to 1.01	0.761 ^a^			
Preoperative UA, (μmol/L), continuous	1.00	0.99 to 1.00	0.098 ^a^			
Preoperative e-GFR, (mL/min/1.73 m^2^), continuous	0.99	0.98 to 1.01	0.452 ^a^			
Preoperative Cystatin-C group						
Preoperative Cystatin-C≤1.09 mg/L	1.00 (ref.)			1.00 (ref.)		
Preoperative Cystatin-C>1.09 mg/L	4.03	2.15 to 7.54	<0.001 ^a^	1.59	1.10 to 2.29	0.012 ^b^

Abbreviation: HR: hazard ratio; CI: confidence interval BMI: body mass index; ALP: alkaline phosphatase; TP: total protein; UA: uric acid; CRE: creatinine; e-GFR: estimated glomerular filtration rate; ref.: reference.

^a^ univariate cox regression analyses

^b^ multivariate cox regression analyses.

**Fig 3:**
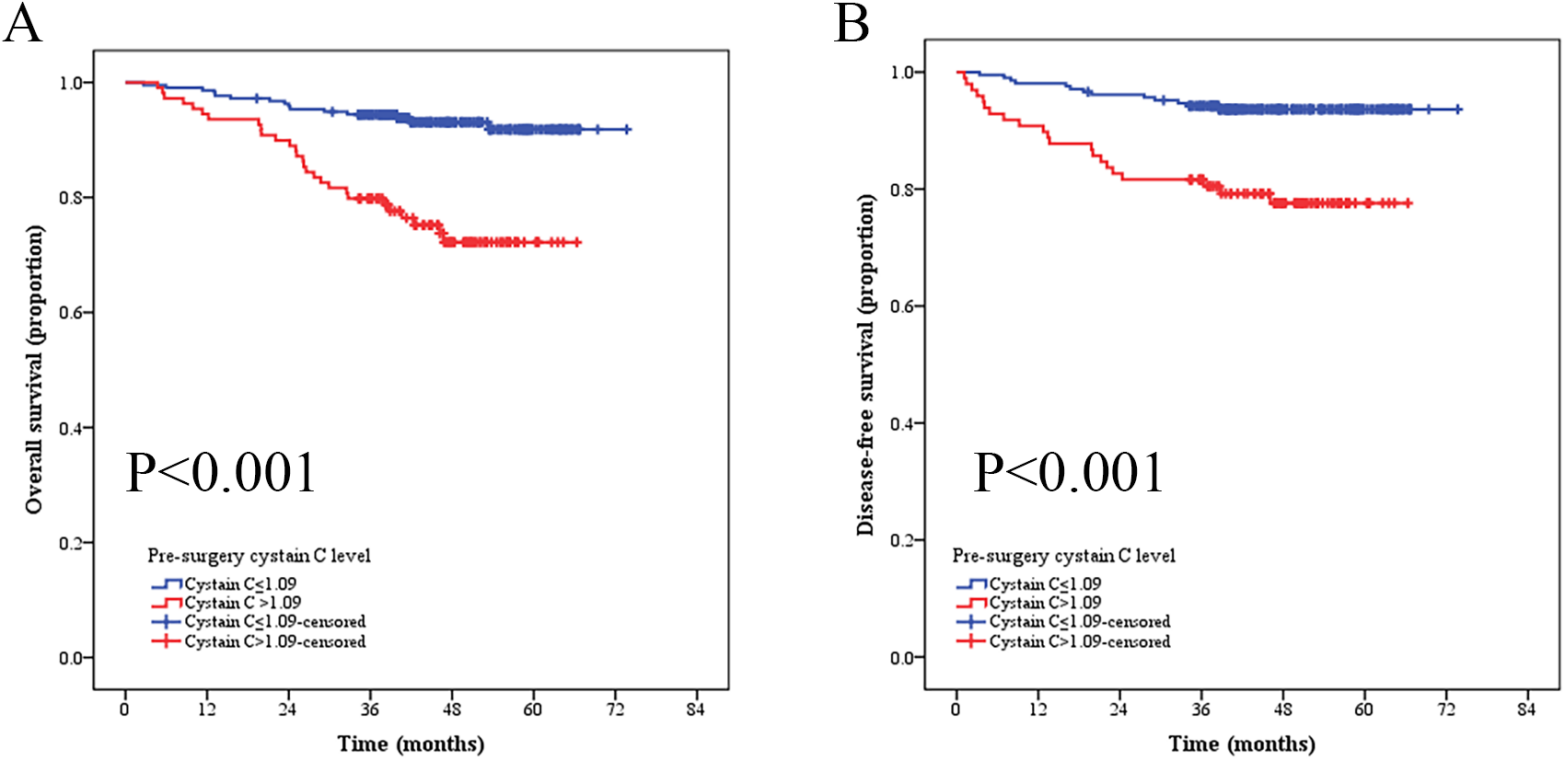
Kaplan-Meier curves depicting overall survival (OS) of disease-free survival (DFS) of (A) 325 patients and (B) 306 patients (M0) with renal cell cancer according to their preoperative cystatin C level.

**Fig 4:**
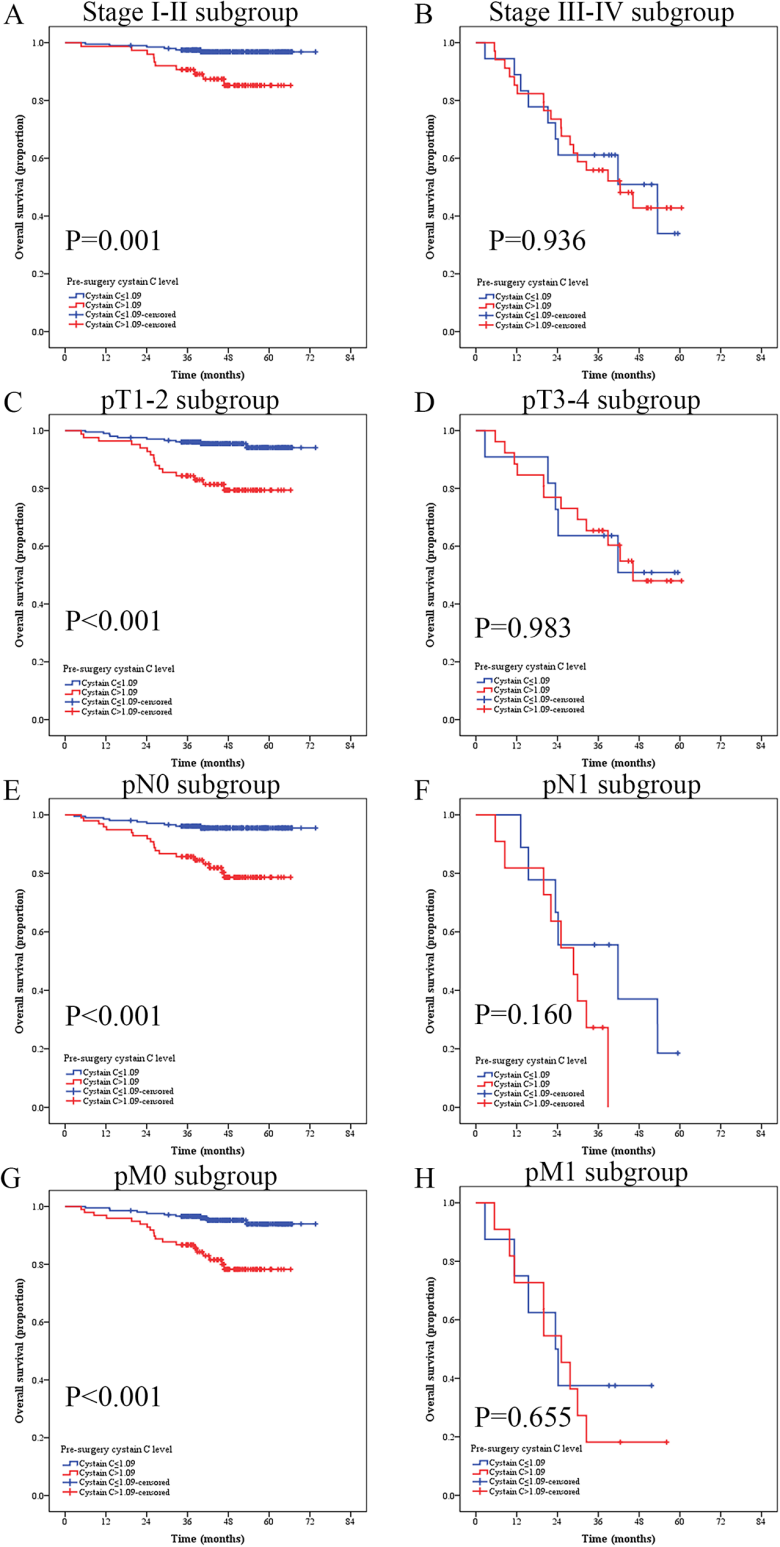
Kaplan-Meier curves depicting overall survival (OS) of 325 patients with renal cell cancer stratified at different pT-status, pN-status, and pM-status according to their preoperative cystatin C level. (A) Patients in I-II subgroup; (B) Patients in III-IV subgroup; (C) Patients in T1-2 subgroup; (D) Patients in T3-4 subgroup; (E) Patients in in N0 subgroup; (F) Patients in N1 subgroup; (G) Patients in M0 subgroup; (H) Patients in M1 subgroup.

To further investigate the prognostic significance of serum Cys-C level in RCC patients, the whole cohort was compared using Kaplan-Meier method and log-rank test. Patients with Cys-C≤1.09 mg/L (n = 216) showed a significantly better OS than patients with serum Cys-C>1.09 mg/L (n = 109) (Cys-C≤1.09 mg/L vs. >1.09 mg/L, mean OS: 69.86 vs. 55.20 months, respectively, P<0.001, **Fig 3A**). We also evaluated the prognostic influence of serum Cys-C level in the subgroups based on the pT-status, pN-status, pM-status, respectively. Patients with low serum Cys-C level had significantly longer OS than compared patients with high serum Cys-C level in the stage I-II subgroup (n = 204, Cys-C≤1.09 mg/L vs. ≥1.09 mg/L, mean OS: 72.10 vs. 60.96 months, respectively, P = 0.001, **Fig 4A**), T1-2 subgroup (n = 288, Cys-C≤1.09 mg/L vs. ≥1.09 mg/L, mean OS: 70.99 vs. 58.12 months, respectively, P<0.001, **Fig 4C**), N0 subgroup (n = 305, Cys-C≤1.09 mg/L vs. >1.09 mg/L, mean OS: 71.27 vs. 57.99 months, respectively, P<0.001, **Fig 4E**), and M0 subgroup (n = 316, Cys-C≤1.09 mg/L vs. >1.09 mg/L, mean OS: 71.13 vs. 58.19 months, respectively, P<0.001, **Fig 4G**). However, the OS was not significantly different in RCC patients at stage III-IV subgroup (n = 52, Cys-C≤1.09 mg/L vs. >1.09 mg/L, mean OS: 39.67 vs. 39.85 months, respectively, P = 0.936, **Fig 4B**), T3-4 subgroup (n = 37, Cys-C≤1.09 mg/L vs. >1.09 mg/L, mean OS: 42.05 vs.42.83 months, respectively, P = 0.983, **Fig 4D**), N1 subgroup (n = 20, Cys-C≤1.09 mg/L vs. >1.09 mg/L, mean OS: 37.09 vs. 26.26 months, respectively, P = 0.160, **Fig 4F**), or M1 subgroup (n = 19, Cys-C≤1.09 mg/L vs. >1.09 mg/L, mean OS: 29.00 vs. 26.72 months, respectively, P = 0.655, **Fig 4H**).

### The relationship of preoperative serum Cys-C with DFS of RCC patients

For DFS, we excluded the patients with pM1 classification (n = 19). 306 patients were enrolled to analyze the relationship of preoperative serum Cys-C with clinicpathologic characteristics. Univariate Cox proportional hazards regression model analysis showed that low serum Cys-C was an independent predictor favorable to DFS (P = 0.001), and BMI, pTNM stage, pT-status, pN-status, and ALP remained clinically and statistically significant predictors of prognosis (**Table 6**). Multivariate analysis was used to adjust the confounders of the association of preoperative serum Cys-C levels with disease progression. Considering the mulicolinearity between pTNM stage and pT-status as well as pN-status, pTNM stage was not included in the multivariate analysis. The results showed that low serum Cys-C was a significantly independent predictor favorable to DFS (HR, 3.50; P = 0.013). In addition, the pN-status was also a clinically and statistically significant predictor of prognosis (**Table 6**).

**Table 6:** Univariate and multivariate analyses in 306 patients with RCC for disease-free survival (DFS).

	Univariate Analysis			Multivariate Analysis		
Variables	HR	95%CI	P value	HR	95%CI	P value
Age, (years) continuous	1.01	0.98 to 1.04	0.457 ^a^			
Gender						
Male	1.00 (ref.)					
Female	0.99	0.48 to 2.04	0.985 ^a^			
BMI	0.84	0.76 to 0.94	0.001 ^a^	0.93	0.81 to 1.05	0.263 ^b^
Fuhrman grade						
Ⅰ	1.00 (ref.)			1.00 (ref.)		
Ⅱ	1.39	0.39 to 4.86	0.610 ^a^	1.04	0.29 to 3.78	0.941 ^b^
Ⅲ	3.80	0.98 to 14.69	0.053 ^a^	2.57	0.58 to 11.41	0.212 ^b^
Ⅳ	18.75	3.76 to 93.52	<0.001 ^a^	21.99	3.92 to 123.29	<0.001 ^b^
unknown	1.79	0.48 to 6.76	0.388 ^a^	0.69	0.15 to 3.11	0.635 ^b^
Pathological types						
Clear cell carcinoma	1.00 (ref.)					
Papillary carcinoma	1.79	0.54 to 5.91	0.338 ^a^			
Others	0.84	0.29 to 2.41	0.750 ^a^			
pTNM-stage						
Ⅰ	1.00 (ref.)					
Ⅱ	2.53	0.95 to 6.75	0.063 ^a^			
Ⅲ	10.95	4.91 to 24.41	<0.001 ^a^			
Ⅳ	15.15	4.87 to 47.1	<0.001 ^a^			
pT-stage						
pT1	1.00 (ref.)			1.00 (ref.)		
pT2	2.68	1.13 to 6.31	0.024 ^a^	1.80	0.67 to 4.83	0.238 ^b^
pT3	6.03	2.46 to 14.81	<0.001 ^a^	1.02	0.32 to 3.20	0.972 ^b^
pT4	12.16	4.03 to 36.75	<0.001 ^a^	2.07	0.47 to 9.01	0.329 ^b^
pN-stage						
pN0	1.00 (ref.)			1.00 (ref.)		
pN1	18.68	8.96 to 38.95	<0.001 ^a^	16.24	to 48.22	<0.001 ^b^
Preoperative ALP, (U/L) continuous	1.01	1.00 to 1.01	<0.001 ^a^	0.99	0.99 to 1.00	0.984 ^b^
Preoperative TP, (g/L), continuous	1.02	0.96 to 1.08	0.515 ^a^			
Preoperative CRE, (μmol/L), continuous	1.01	0.99 to 1.02	0.221 ^a^			
Preoperative UA, (μmol/L), continuous	1.00	0.99 to 1.00	0.415 ^a^			
Preoperative e-GFR, (mL/min/1.73 m^2^), continuous	0.99	0.97 to 1.01	0.179 ^a^			
Preoperative Cystatin-C group						
Preoperative Cystain-C≤1.09 mg/L	1.00 (ref.)			1.00 (ref.)		
Preoperative Cystatin-C>1.09 mg/L	3.76	1.88 to 7.51	<0.001 ^a^	3.50	1.29 to 9.51	0.013 ^b^

Abbreviation: HR: hazard ratio; CI: confidence interval BMI: body mass index; ALP: alkaline phosphatase; TP: total protein; UA: uric acid; CRE: creatinine; e-GFR: estimated glomerular filtration rate; ref.: reference.

^a^ univariate cox regression analyses

^b^ multivariate cox regression analyses.

Kaplan-Meier method and log-rank test were used to compare the different effects of serum Cys-C level on DFS. Patients with serum Cys-C ≤1.09 mg/L (n = 198) showed a significantly better DFS than patients with serum Cys-C>1.09 mg/L (n = 75) (Cys-C ≤1.09 mg/L vs. >1.09 mg/L, mean DFS: 71.95 vs. 58.10 months, respectively, P<0.001, **Fig 3B**).

**Fig 5:**
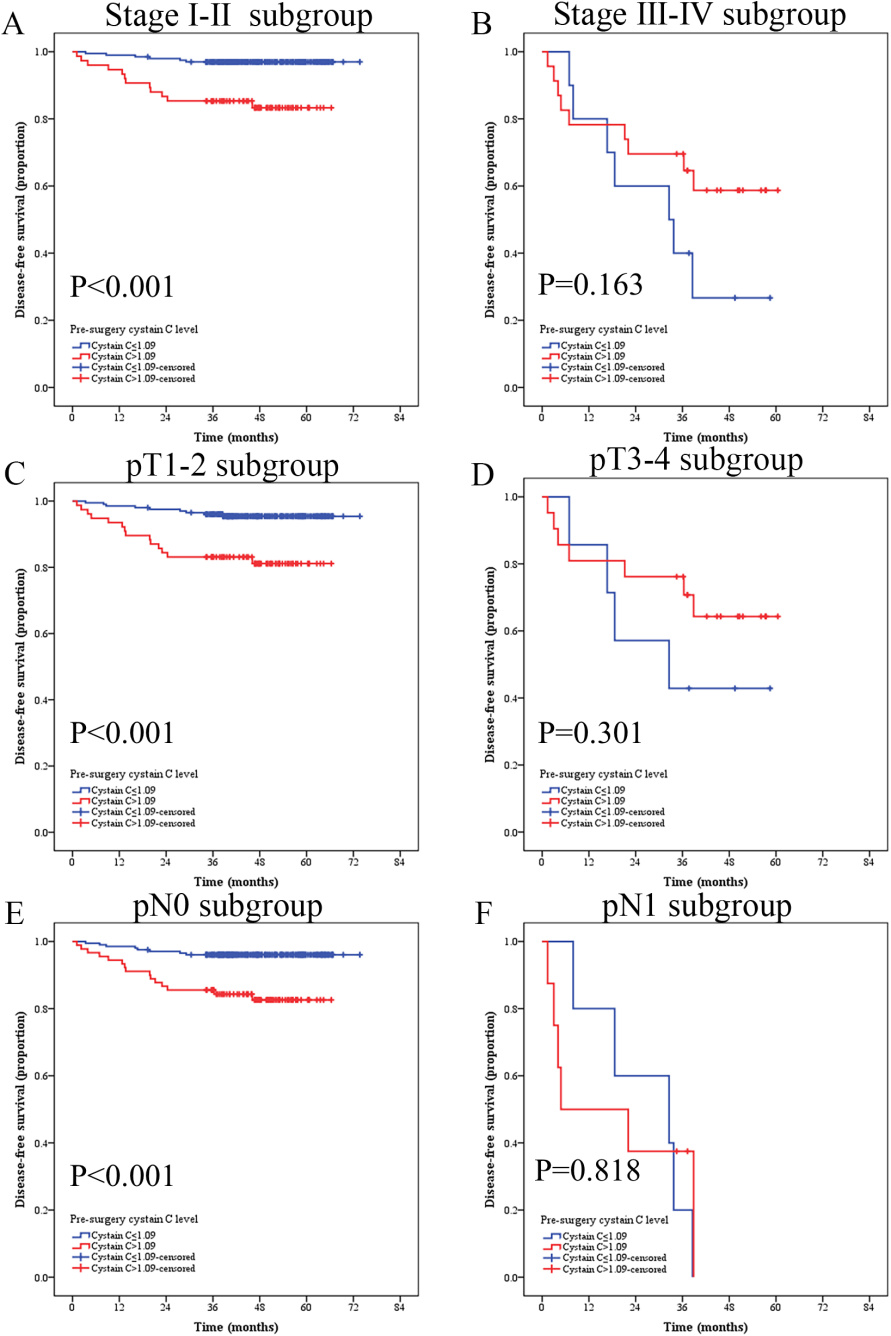
Kaplan-Meier curves depicting disease-free survival (DFS) according to preoperative cystatin C levels in 306 patients with renal cell cancer. **Patients were stratified according to the pT-status, pN-status, and pM-status.** (A) Kaplan-Meier analysis of DFS in Ⅰ-Ⅱsubgroup; (B) Kaplan-Meier analysis of DFS in III-Ⅳsubgroup. (C) Kaplan-Meier analysis of DFS in T1-2 subgroup. (D) Kaplan-Meier analysis of DFS in T3-4 subgroup. (E) Kaplan-Meier analysis of DFS in N0 subgroup. (F) Kaplan-Meier analysis of DFS in N1 subgroup.

We compared survival curves of patients with low and high preoperative serum Cys-C at different pathological stages (pT-status, pN-status, pM-status) using Kaplan-Meier method and log-rank test. In stage I-II subgroup, patients with high serum Cys-C had a significantly poorer DFS than patients with low serum Cys-C patients **(**n = 273, Cys-C≤1.09 mg/L vs. >1.09 mg/L, mean OS: 71.95 vs. 58.10 months, respectively, P<0.001, **Fig 5A)**. In pT1-2 subgroup, patients with high serum Cys-C also had a significantly poorer DFS than patients with low serum Cys-C patients **(**n = 278, Cys-C≤1.09 mg/L vs. >1.09 mg/L, mean OS: 71.24 vs. 56.94 months, respectively, P<0.001, **Fig 5C)**. In the pN0 subgroup, patients with high serum Cys-C had poorer DFS than patients with low serum Cys-C **(**n = 293, Cys-C≤1.09 mg/L vs. >1.09 mg/L, mean OS: 71.38 vs. 57.93 months, respectively, P<0.001, **Fig 5E)**. However, there was no significance difference in DFS in stage III-IV **(**n = 33, Cys-C≤1.09 mg/L vs. >1.09 mg/L, mean OS: 32.38 vs. 42.36 months, respectively, P = 0.163, **Fig 5B)**, pT3-4 n = 28, Cys-C≤1.09 mg/L vs. >1.09 mg/L, mean OS: 35.74 vs. 45.12 months, respectively, P = 0.301 **Fig 5D)**, and pN1 **(**n = 13, Cys-C≤1.09 mg/L vs. >1.09 mg/L, mean OS: 26.28 vs. 18.99 months, respectively, P = 0.818, **Fig 5F)** groups.

## Discussion

This is the first study to analyze the relationship between preoperative serum Cys-C and the prognosis on RCC. Previous studies had found that elevated serum Cys-C is associated with poor prognosis of other cancer patients [16]. However, no study has explored to the prognostic value of serum Cys-C on RCC patients. Patients with preoperative high serum Cys-C (>1.09 mg/L) had shorter OS and DFS than patients with low serum Cys-C (≤1.09 mg/L). The results of multivariate analysis demonstrated that preoperative serum Cys-C was an independent factor for predicting OS of RCC patients. In addition, preoperative serum Cys-C was also an independent factor for predicting DFS of non-metastatic RCC patients treated with complete surgical resection.

Analyses of relationship between serum Cys-C level and OS and DFS of patients at different stages (pTNM-stage, pT-stage, pN-stage and pM-stage) demonstrated that low preoperative serum Cys-C level was a favorable prognostic factor for OS of patients at pT1-2, pN0 and pM0 stages and DFS of patients at pT1-2 and pN0 stages, but had no statistically significant association with OS of patients at pT3-4, pN1 and pM1 stages and DFS of patients at pT3-4 and pN1 stages, probably due to insufficient number of patients at advanced stages.

As a member of cysteine protease inhibitors, Cys-C inhibits tumor invasion and metastasis. Downregulation of Cys-C is frequently reported in breast, prostate, stomach, uterus, colon, and non-small cell lung cancer tissues [19–22], but controversial in other cancers [23, 24]. Cys-C level is rarely detected or reduced in tumor tissues of RCC patients comparing that in normal tissues [25]. Overall, Cys-C expression in cancer tissues indicates that Cys-C expression is primarily suppressed by tumorigenesis and decreased Cys-C level may induce tumor formation, invasion and metastasis.

Circulating Cys-C is also considered to function as a tumor suppressor [26]. However, contrast to the reduced Cys-C level in cancer tissues, serum Cys-C level is elevated in cancer patients and associated with their poor prognosis [27, 28]. In this study, we showed for the first time that serum Cys-C is elevated in RCC patients at pT3-4 stage than at pT1-2 stage, and in RCC patients at pM1 stage than at pM0 stage (**Fig 2**). Moreover, patients with high serum Cys-C level (>1.09 mg/L) at pT1-2, pN0 and pM0 stages had worse OS than patients with low serum Cys-C level (≤1.09 mg/L) at pT1-2, pN0 and pM0 stages, suggesting that serum Cys-C level could be a good prognostic biological marker for RCC patients.

As the major substrate of Cys-C, Cysteine cathepsin protease activity is frequently dysregulated in the context of neoplastic transformation [29, 30]. Increased activity and aberrant localization of proteases in tumor microenvironment have a potent role in driving cancer progression, proliferation, invasion and metastasis [31]. The elevated cathepsin expression is significantly associated with poor prognosis of patients with melanoma as well as breast, lung, head and neck, colorectal and many other cancers [32–34]. It has been shown that cathepsin D expression level was higher in RCC tumor tissues and urine than benign or normal volunteer samples [35, 36]. However, serum cathepsin D is not altered in RCC patients in comparison with healthy volunteers [37] and cathepsins B, C, H, L and S are not higher in RCC tissue than in normal kidney [38]. Although changes of cathepsins expression in RCC are controversial, the balance between Cys-C and its substrates palain-like cysteine proteases is very important for tumor cell invasion and metastasis. Elevated serum Cys-C in RCC may be a reflection of increased level and activity of extracellular proteases (such as palain-like cysteine proteases and other EMC proteases) in tumors or the stromal host cells [39]. Therefore, the relation of RCC at high stage with increased serum Cys-C may be due to its more aggressive characteristics and higher proteases level. Elevated serum Cys-C level of cancer patients is not directly associated with the invasion of tumor cells into target organs, but represents a secondary effect due to a reduced elimination rate of Cys-C by glomeruli caused by disease-related kidney damages [40]. For example, higher stage RCC may damage more renal structure and function, therefore reducing GFR and increasing serum Cys-C.

Previous studies demonstrated Serum Cys-C is an important biomarker of renal function and associated better with direct measures of glomerular filtration rate more precisely than CRE and e-GFR, because serum CRE is dependent not only on GFR, but also on body muscle mass, which are affected by age and sex, and the e-GFR in our study is calculated using creatinine clearance using serum CRE levels [41, 42]. Estimation of renal function is important since renal insufficiency is directly associated with increased mortality after cancer [43]. To explore the effect of renal function on prognosis, we also analyzed the association of serum Cys-C, CRE and e-GFR with the prognosis of RCC patients, respectively. Our results showed that serum Cys-C was a better prognostic indicator than CRE and e-GFR and that preoperative serum Cys-C rather than CRE and e-GFR is the prognostic factor for OS of RCC patients after surgery. Although some studies showed that end-stage renal disease would affect tumor prognosis, the association of early renal injury and cancer prognosis is not clear [44]. Our results showed that no patient died of end-stage renal disease, most possibly due to short follow-up time. In addition, our resulted also showed that preoperative serum Cys-C level was positively correlated with CRE, but negatively correlated with e-GFR. Therefore, regardless of the loss of renal function by renal structure destroy by tumor mass, serum Cys-C is still a predictor of RCC although the role of serum Cys-C in RCC progression needs to be further explored.

The study has some limitations. Firstly, it is a retrospective, single-center study, which may limit the prognostic value of serum Cys-C. Therefore, a large-scale prospective validation study is needed. Secondly, Cys-C differs among individuals and can be influenced by heterogeneity in the treatment used for the RCC patients after surgical resection, which led to different clinical prognosis; such therapeutic strategy should be considered in the future analysis. Thirdly, as the survival of early stage RCC patients is greatly extended, longer following time is needed to obtain a more reliable result. Lastly, our results still need multicenter validation.

## Conclusions

In conclusion, our results revealed that preoperative serum Cys-C level could be considered as not only the renal function predictor, but also an independent prognostic factor for surgical RCC patients. This is also confirmed in RCC patients at early stages according to pT, pN and pM classifications. Overall, serum Cys-C may be a convenient and useful biomarker to distinguish RCC patients with high risk of recurrent or metastasis after surgery and RCC patients with higher serum Cys-C level should be more closely followed up.

## Ethical approval

This retrospective study was approved by the Institute Research Medical Ethics Committee of Sun Yat-sen University Cancer Center. All procedures performed in studies involving human participants were in accordance with the ethical standards of the institutional and/or national research committee and with the 1964 Declaration of Helsinki and its later amendments or comparable ethical standards. All included patients provided written informed consent and their information were recorded and registered in our cancer registry system.

## Supporting information

S1 Table Relevant data underlying the study. (XLSX)

## Abbreviations

RCC: Renal cell carcinoma
Cys-C: Cystatin-C
e-GFR: Estimated glomerular filtration rate
BMI: Body mass index
ALP: Alkaline phosphatase
LDH: Lactate dehydrogenase
CRE: Serum creatinine
TP: Total protein
UA: Uric acid
